# Race, Ethnicity, and Delayed Time to COVID-19 Testing Among US Health Care Workers

**DOI:** 10.1001/jamanetworkopen.2024.5697

**Published:** 2024-04-10

**Authors:** DaMarcus E. Baymon, J. Priyanka Vakkalanka, Anusha Krishnadasan, Nicholas M. Mohr, David A. Talan, Melissa Briggs Hagen, Kelli Wallace, Karisa K. Harland, Imoigele P. Aisiku, Peter C. Hou

**Affiliations:** 1 Department of Emergency Medicine, Brigham and Women’s Hospital, Boston, Massachusetts; 2 Department of Emergency Medicine, Carver College of Medicine, University of Iowa, Iowa City; 3 Department of Epidemiology, College of Public Health, University of Iowa, Iowa City; 4 Olive View-University of California, Los Angeles Education and Research Institute, Los Angeles; 5 Department of Anesthesia Critical Care, Carver College of Medicine, University of Iowa, Iowa City; 6 David Geffen School of Medicine, University of California, Los Angeles; 7 National Center for Immunizations and Respiratory Diseases, Centers for Disease Control & Prevention, Atlanta, Georgia; 8 University of Iowa Holden Comprehensive Cancer Center, University of Iowa, Iowa City

## Abstract

**Importance:**

Access to COVID-19 testing is critical to reducing transmission and supporting early treatment decisions; when made accessible, the timeliness of testing may also be an important metric in mitigating community spread of the infection. While disparities in transmission and outcomes of COVID-19 have been well documented, the extent of timeliness of testing and the association with demographic factors is unclear.

**Objectives:**

To evaluate demographic factors associated with delayed COVID-19 testing among health care personnel (HCP) during the COVID-19 pandemic.

**Design, Setting, and Participants:**

This cross-sectional study used data from the Preventing Emerging Infections Through Vaccine Effectiveness Testing study, a multicenter, test-negative, case-control vaccine effectiveness study that enrolled HCP who had COVID-19 symptoms and testing between December 2020 and April 2022. Data analysis was conducted from March 2022 to Junne 2023.

**Exposure:**

Displaying COVID-19–like symptoms and polymerase chain reaction testing occurring from the first day symptoms occurred up to 14 days after symptoms occurred.

**Main Outcomes and Measures:**

Variables of interest included patient demographics (sex, age, and clinical comorbidities) and COVID-19 characteristics (vaccination status and COVID-19 wave). The primary outcome was time from symptom onset to COVID-19 testing, which was defined as early testing (≤2 days) or delayed testing (≥3 days). Associations of demographic characteristics with delayed testing were measured while adjusting for clinical comorbidities, COVID-19 characteristics, and test site using multivariable modeling to estimate relative risks and 95% CIs.

**Results:**

A total of 5551 HCP (4859 female [82.9%]; 1954 aged 25-34 years [35.2%]; 4233 non-Hispanic White [76.3%], 370 non-Hispanic Black [6.7%], and 324 non-Hispanic Asian [5.8%]) were included in the final analysis. Overall, 2060 participants (37.1%) reported delayed testing and 3491 (62.9%) reported early testing. Compared with non-Hispanic White HCP, delayed testing was higher among non-Hispanic Black HCP (adjusted risk ratio, 1.18; 95%CI, 1.10-1.27) and for non-Hispanic HCP of other races (adjusted risk ratio, 1.17; 95% CI, 1.03-1.33). Sex and age were not associated with delayed testing. Compared with clinical HCP with graduate degrees, all other professional and educational groups had significantly delayed testing.

**Conclusions and Relevance:**

In this cross-sectional study of HCP, compared with non-Hispanic White HCP and clinical HCP with graduate degrees, non-Hispanic Black HCP, non-Hispanic HCP of other races, and HCP all other professional and education backgrounds were more likely to have delayed COVID-19 testing. These findings suggest that time to testing may serve as a valuable metric in evaluating sociodemographic disparities in the response to COVID-19 and future health mitigation strategies.

## Key Points

**Question:**

Does time from symptom onset to COVID-19 testing among health care personnel (HCP) differ by race and ethnicity, sex, and age?

**Findings:**

In this cross-sectional study, delayed testing was higher among non-Hispanic Black HCP and non-Hispanic HCP of other races compared with non-Hispanic White HCP. There were no significant differences in age or sex for COVID-19 testing after symptom onset.

**Meaning:**

These findings suggest that future investigations into the systemic factors or barriers contributing to race and ethnicity differences in COVID-19 testing after symptom onset must be explored in an effort to minimize COVID-19 spread.

## Teaser

This cross-sectional study analyzes demographic factors associated with delayed COVID-19 testing among health care personnel.

## Introduction

COVID-19 was declared a pandemic by the World Health Organization in March 2020 due to its extensive spread and rising global prevalence.^1^ Since the pandemic started, there have been substantial disparities reported in COVID-19 infection rates and severe clinical outcomes (eg, hospitalization, admission to intensive care units, ventilation, and death).^2,3,4^ These findings have documented how individual-level demographics such as age, race, ethnicity, and underlying clinical comorbidities are associated with higher infection rates and worse clinical outcomes.^2,3,4,5^

In addition to demographic characteristics, inequities in COVID-19 outcomes have also been attributed to disparities in access to health care, testing, vaccination, and COVID-19 treatments; local societal norms and culture; and systemic bias.^6,7,8^ COVID-19 testing was initially a challenge because the availability of tests were limited.^9^ Once testing was made more widely available, the timeliness of testing subsequently became a critical factor; identification of a positive test allows for modification of behaviors (eg, isolation and quarantine), contact tracing, and receipt of treatment as part of effective public health strategies for reducing community-level spread of the infection and improving population-based outcomes.^10,11^

In this study, we investigated the timeliness of COVID-19 testing in a population that had access to employer-based testing. We aimed to assess whether demographic variables such as sex, age, race, and ethnicity were associated with delays in testing in a cohort of health care personnel (HCP) who had symptoms consistent with COVID-19. Our goal was to understand whether disparities related to timeliness of COVID-19 testing exist because timely testing could help with the mitigation and management of the COVID-19 epidemic and future emergent pathogens of global public health concern.

## Methods

### Study Design, Setting, and Sample

This cross-sectional study was determined to not be human participants research but rather public health surveillance by the Centers for Disease Control and Prevention (CDC) institutional review board, and all site institutional review boards agreed with the designation of public health surveillance activity and used deidentified data in accordance with the Common Rule. The study followed the Strengthening the Reporting of Observational Studies in Epidemiology (STROBE) reporting guideline.^14^ This study analyzed testing practices in participants enrolled in the Preventing Emerging Infections Through Vaccine Effectiveness Testing (PREVENT) project,^12^ a test-negative, case-control study of HCP enrolled across 15 academic medical centers in the US. Details of the PREVENT study design have been described previously.^12^ Briefly, participants were HCP who were symptomatic with and without SARS-CoV-2 infection selected because they exclusively had polymerase chain reaction–based COVID-19 testing outside of the home setting between December 2020 and April 2022. We included participants with a COVID-19 test occurring from the first day symptoms occurred (ie, day 0) up to 14 days after symptoms occurred. Exclusion criteria analysis included COVID-19 tests that had indeterminate results or were unverified by local site personnel, tests that were performed prior to any symptom onset, tests occurring more than 14 days after symptom onset, and those testing for reasons other than for COVID-19–like symptoms (eg, routine testing). Testing activity was reviewed and conducted in accordance with applicable federal law and CDC policy.^13^ All HCP provided written informed consent before participation.

### Outcome

Our outcome was time to COVID-19 testing, which we dichotomized as delayed (testing first performed ≥3 days after the onset of symptoms) or early (testing first performed ≤2 days after the onset of symptoms). This cutoff point was determined by evaluating the distribution of time to testing and consensus within the study team to identify cases that might have had the opportunity for earlier testing within participating medical centers (eg, considering test availability on weekends).

### Primary Exposures

Our primary exposures of interest in this study were demographic characteristics of HCP including sex (ie, male or female), age (ie, 18-24, 25-34, 35-49, and 50-64 years), race, and ethnicity. Options for race and ethnicity allowed participants to self-select multiple categories, and we categorized this variable as Hispanic White, non-Hispanic Asian, non-Hispanic Black, non-Hispanic White, Hispanic and other race (ie, Hispanic ethnicity and American Indian or Alaskan Native, Asian, Black, Native Hawaiian or Pacific Islander, or multiple combinations of race), and missing ethnicity (ie, includes Asian, Black, Native Hawaiian or Pacific Islander, or no race selection).

### Secondary Covariates and Confounders

Other demographic covariates in this study included education level, job classification, and health insurance (private, government, none, or other). We combined education and job classification to indicate education (eg, high school or less, some college or undergraduate education, technical degree, and graduate or professional degree) and job classification (clinical [eg, nurse or nurse assistant, physician, advanced practice clinician, housekeeping, or other clinical position] vs nonclinical [eg, research staff, security, or laboratory personnel]) together.

Clinical characteristics assessed included number of comorbidities identified at baseline (≥3 or ≤2), specific conditions (ie, hypertension, diabetes, immunosuppressing conditions, and asthma), body mass index (BMI; calculated as weight in kilograms divided by height in meters squared) category, and pregnancy status. COVID-19–related characteristics included vaccination status and COVID-19 variant wave. Vaccination status was classified as no documentation of vaccines, partial or non–mRNA vaccination (eg, 1 dose of mRNA vaccine or single dose of a non-mRNA vaccine), or completed primary series (defined as 2 doses of mRNA vaccine administered more than 2 weeks apart and prior to the index COVID-19 test). Additional vaccines, including boosters, were not considered at this time. COVID-19 variant waves were defined using CDC date-based assignment for the predominant circulating strain, with test dates prior to June 19, 2021, categorized as the wild type or Alpha variant wave; those between June 20, 2021, and December 18, 2021, as the Delta variant wave; and the rest of the study period as the Omicron variant wave.^15^

### Statistical Analysis

We assessed the distributions of demographic, clinical, and situational characteristics by each outcome category. We built bivariate generalized estimating equations using a log link and binomial distribution to evaluate the relative risk (RR) of delayed compared with early testing and corresponding 95% CIs using complete case analysis. Final models included demographics (sex, age group, race and ethnicity, and education and job classification), clinical history characteristics (number of comorbidities, diabetes, hypertension, smoking status, and BMI), COVID-19 characteristics (COVID-19 wave and vaccination status), and study site.

No sample size was calculated for this analysis because we used the entire multicenter sample of eligible participants in the PREVENT study.^12^ All analyses were completed using SAS statistical software version 9.5. Data analysis occurred from March 2022 to June 2023.

## Results

### Characteristics of Study Participants

**Figure 1.**
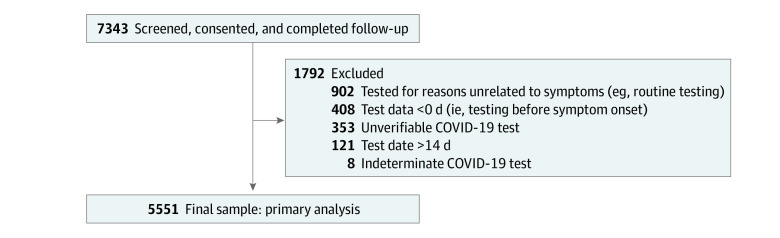
Flowchart of Final Sample

Of the 7343 HCP who completed follow-up in the PREVENT study, 5551 (1954 aged 25-34 years [35.2%]; 4859 female [82.9%]; 4233 non-Hispanic White [76.3%]; 370 non-Hispanic Black [6.7%]; 324 non-Hispanic Asian [5.8%]) were included in this analysis (Figure 1 and Table). Most participants had 2 or fewer comorbidities (4686 participants [84.4%]). Of the 5551 participants, 1105 (19.9%) had a clinical job classification with a graduate degree; 1558 (28.1%) had a clinical role with some college education, a college degree, or technical degree; 1028 (18.5%) were nonclinical HCPs with a graduate degree; 1674 (30.1%) were nonclinical HCP with some college education, a college degree, or a technical degree; 185 (3.3%) were nonclinical HCP with a high school education or less; and educational and job information was unknown for 4 participants (0.1%). Of all participants, 4142 (74.6%) were fully vaccinated with the primary series at the time of their illness.

**Table.** Demographic and Clinical Characteristics of Health Care Personnel by Early vs Late COVID-19 Testing, December 2020 to May 2022

Demographic and clinical characteristics	Participants No. (%) (N = 5551)^b^	Time to any first symptom, No. (%)^a^		Unadjusted relative risk (95% CI)
		≥3 d to test (n = 2060)	≤2 d to test (n = 3491)	
Sex				
Female	4589 (82.9)	1726 (37.6)	2861 (62.4)	1 [Reference]
Male	954 (17.2)	333 (34.9)	621 (65.1)	0.93 (0.54-1.02)
Age group, y				
18-24	1230 (22.2)	456 (37.1)	774 (62.9)	1 [Reference]
25-34	1954 (35.2)	701 (35.9)	1253 (64.1)	0.97 (0.88-1.06)
35-49	1134 (20.4)	411 (36.2)	723 (63.8)	0.98 (0.88-1.09)
50-64	1233 (22.2)	492 (39.9)	741 (60.1)	1.08 (0.97-1.19)
Race and ethnicity				
Hispanic White	300 (5.4)	116 (38.7)	184 (61.3)	1.24 (1.02-1.50)
Hispanic other^c^	125 (2.3)	56 (44.8)	69 (55.2)	1.24 (1.02-1.50)
Non-Hispanic Asian	324 (5.8)	114 (35.2)	210 (64.8)	0.97 (0.83-1.14)
Non-Hispanic Black	370 (6.7)	167 (45.1)	203 (54.9)	1.25 (1.11-1.40)
Non-Hispanic White	4233 (76.3)	1530 (36.1)	2703 (63.9)	1 [Reference]
Non-Hispanic Other^d^	174 (3.1)	72 (41.4)	102 (58.6)	1.14 (0.95-1.37)
Unknown ethnicity^e^	25 (0.5)	5 (20.0)	20 (80.0)	0.55 (0.21-1.43)
Health insurance				
Private	5138 (92.6)	1892 (36.8)	3246 (63.2)	1 [Reference]
Government	170 (3.1)	70 (41.2)	100 (58.8)	1.12 (0.93-1.34)
None	17 (0.3)	4 (23.5)	13 (76.5)	0.64 (0.24-1.70)
Other	222 (4.0)	92 (41.4)	130 (58.6)	1.13 (0.96-1.32)
Job classification and education				
Clinical: graduate degree	1105 (19.9)	338 (30.6)	767 (69.4)	1 [Reference]
Clinical: some college, college degree, or technical degree	1558 (28.1)	597 (38.3)	961 (61.7)	1.25 (1.12-1.40)
Nonclinical: graduate degree	1028 (18.5)	374 (36.1)	657 (63.9)	1.18 (1.04-1.34)
Nonclinical: some college, college degree, or technical degree	1674 (30.1)	667 (39.9)	1004 (60.1)	1.31 (1.17-1.46)
High school or less	185 (3.3)	86 (46.5)	99 (53.5)	1.52 (1.27-1.81)
Unknown	4 (0.1)	1 (25.0)	3 (75.0)	0.82 (0.12-5.41)
Comorbidities and clinical presentation				
No. of comorbidities^f^				
≥3	865 (15.6)	377 (46.6)	488 (56.4)	1.21 (1.12-1.32)
≤2	4686 (84.4)	1683 (35.9)	3003 (64.1)	1 [Reference]
Immunosuppressing condition				
Yes	49 (0.9)	20 (40.8)	29 (59.2)	1.10 (0.79-1.54)
No	5502 (99.1)	2040 (99.0)	3462 (62.9)	1 [Reference]
Asthma				
Yes	861 (15.5)	357 (41.5)	504 (58.5)	1.14 (1.05-1.25)
No	4690 (84.5)	1703 (36.3)	2987 (63.7)	1 [Reference]
Diabetes (type 1 or 2)				
Yes	212 (3.8)	79 (37.3)	133 (62.7)	1.00 (0.84-1.20)
No	5339 (96.2)	1981 (37.1)	3358 (62.9)	1 [Reference]
Hypertension				
Yes	746 (13.4)	293 (39.3)	453 (60.7)	1.07 (0.97-1.18)
No	4805 (86.6)	1767 (36.8)	3038 (63.2)	1 [Reference]
Smoking status				
Never smoked	4234 (76.3)	1541 (36.4)	2693 (63.6)	1 [Reference]
Current smoker	311 (5.6)	120 (38.6)	191 (61.4)	1.06 (0.92-1.23)
Former smoker	819 (14.8)	309 (37.7)	510 (62.3)	1.04 (0.94-1.14)
Prefer not to answer	187 (3.4)	90 (48.1)	97 (51.9)	1.32 (1.14-1.54)
Pregnant				
Yes	125 (2.3)	50 (40.0)	75 (60.0)	2.10 (1.40-3.16)
No	5426 (97.7)	2003 (37.0)	3414 (64.0)	1 [Reference]
Body mass index category^g^				
Healthy (18.5-24.9)	1875 (33.8)	632 (33.7)	1243 (66.3)	1 [Reference]
Underweight (≤18.5)	53 (1.0)	21 (39.6)	32 (60.4)	1.18 (0.84-1.64)
Overweight (25-29.9)	1671 (30.1)	638 (38.2)	1033 (61.8)	1.13 (1.04-1.24)
Obese (≥30)	1952 (35.2)	769 (39.4)	1183 (60.6)	1.17 (1.07-1.27)
COVID-19 characteristics				
Variant				
Alpha	1385 (25.0)	557 (40.2)	828 (59.8)	1 [Reference]
Delta	1860 (33.5)	728 (39.1)	1132 (60.9)	0.97 (0.89-1.06)
Omicron	2306 (41.5)	775 (33.6)	1531 (55.4)	0.84 (0.77-0.91)
Vaccination (2 doses)				
No	701 (12.6)	299 (42.7)	402 (57.4)	1 [Reference]
Yes	4142 (74.6)	1506 (36.4)	2636 (63.6)	0.85 (0.78-0.94)
Incomplete	702 (12.8)	255 (36.0)	453 (64.0)	0.84 (0.74-0.96)

^a^ Represents row percentages.

^b^ Represents column percentages.

^c^ Hispanic-Other includes Hispanic–American Indian or Alaska Native (14 participants), Hispanic-Asian (5 participants), Hispanic-Black (23 participants), Hispanic–Native Hawaiian or Pacific Islander (3), Hispanic and multiple races (23 participants), or Hispanic ethnicity and no race checked (57 participants).

^d^ Non-Hispanic Other includes non-Hispanic American Indian or Alaska Native (17 participants), non-Hispanic Native Hawaiian or Pacific Islander (10 participants), non-Hispanic and multiple races (127 participants), or non-Hispanic ethnicity and no race checked (20 participants).

^e^ Unknown ethnicity was defined as those with unknown ethnicity who selected a specified race category including Asian (3 participants), Black (1 participant), White (18 participants), multiple races (1 participant), or no race checked (2 participants).

^f^ No. of comorbidities is summarized from the following list: immunosuppressing condition, active cancer, alcohol use disorder, allergic rhinitis, anxiety or trauma, asthma, autoimmune condition, coronary artery disease, chronic liver disease, chronic kidney disease, chronic obstructive pulmonary disease, cognitive or neurodevelopmental disorder, deep vein thrombosis, depression, diabetes (type 1 or 2), dialysis, hypertension, movement or motor disorders, sleep disorder, stroke, transplant, other heart conditions, other chronic lung disease, or other mental health conditions.

^g^ Body mass index was calculated as weight in kilograms divided by height in meters squared.

### Time to Testing Characteristics

**Figure 2.**
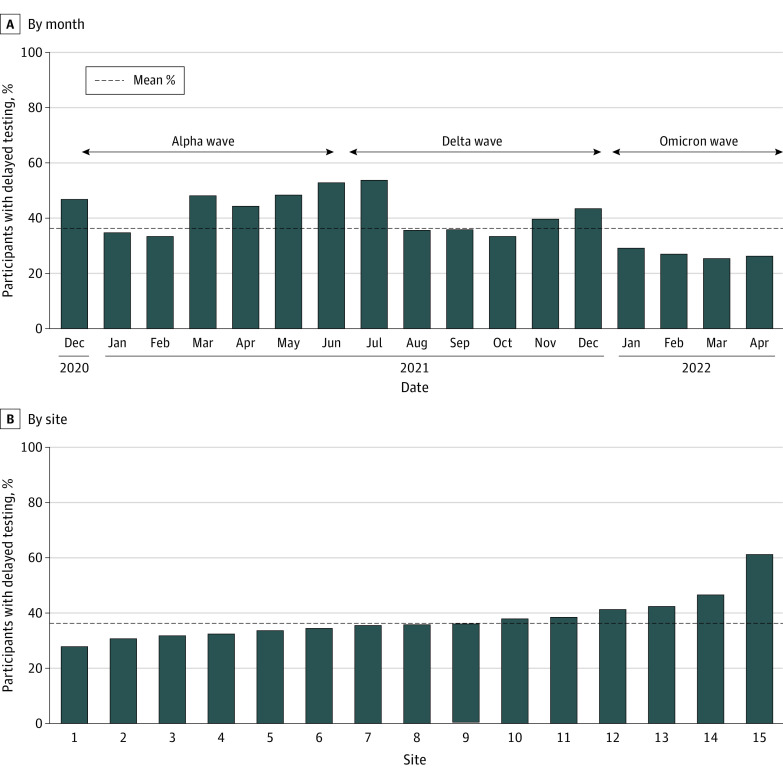
Distribution of Delayed Testing by Testing Month and Participating Site

The majority of HCP (5262 participants [94.8%]) tested within 1 week of symptom onset (median [IQR], 2 [1-3] days) (eFigure in Supplement 1). Overall, 3491 participants (62.9%) were tested early (≤2 days) and 2060 (37.1%) had delayed testing (≥3 days). The proportion of delayed testing ranged by month (24 of 94 participants [25.5%] to 140 of 259 participants [54.1%]) (Figure 2A) and site (18 of 65 participants [27.7%] to 22 of 36 participants [61.1%]) (Figure 2B).

### Delayed Testing Characteristics

There was no association of gender and age with delayed testing (Table). Compared with non-Hispanic White HCP, the likelihood of delayed testing was greater among non-Hispanic Black HCP (unadjusted RR [uRR], 1.25; 95% CI, 1.11-1.40) and Hispanic participants of other races (uRR, 1.24; 95% CI, 1.02-1.50). Compared with clinical HCP with graduate degrees, all other groups were more likely to have delayed testing. Clinical factors associated with delayed testing included those with 3 or more comorbid conditions (uRR, 1.21; 95% CI, 1.12-1.32), asthma (uRR, 1.14; 95% CI, 1.05-1.25), and obesity (uRR, 1.17; 95% CI, 1.07-1.27).

Compared with the Alpha wave, delayed testing was not different in the Delta wave (uRR, 0.97; 95% CI, 0.89-1.06) but was lower in the Omicron wave (uRR, 0.84; 95% CI, 0.77-0.91). Compared with those who were not vaccinated, delayed testing was lower in both incomplete (uRR, 0.84; 95% CI, 0.74-0.96) and fully vaccinated HCP (uRR, 0.85; 95% CI, 0.78-0.94).

**Figure 3.**
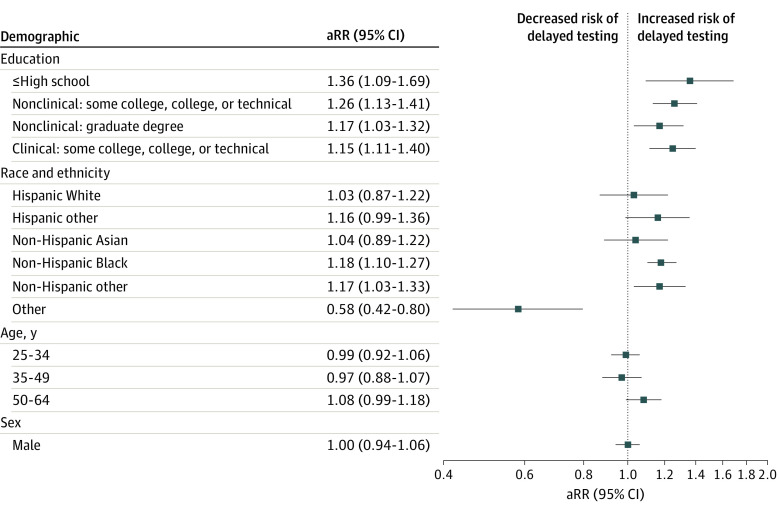
Risk of Early vs Late Testing for COVID-19 Reference groups include having a clinical job with a graduate degree for education, non-Hispanic White for race, 18 to 24 years for age, and female for sex. aRR indicates adjusted relative risk.

In our final multivariable model (Figure 3), the risk of delayed testing was higher in non-Hispanic Black HCP (adjusted RR [aRR], 1.18; 95% CI, 1.10-1.27) and non-Hispanic HCP of other races (aRR, 1.17; 95% CI, 1.03-1.33) compared with non-Hispanic White HCP. Sex and age were not associated with delayed testing. Compared with graduate-level, trained, clinical HCPs, delayed testing was greater among clinical HCPs with some college education, a college degree, or technical degree (aRR, 1.25; 95% CI, 1.11-1.40) and nonclinical HCPs with graduate-level training (aRR, 1.26; 95% CI, 1.13-1.41). Those with an educational background of high school or less (whether clinical or nonclinical) had a higher risk of delayed testing compared with graduate-level, trained, clinical HCPs (aRR, 1.36; 95% CI, 1.09-1.69). The associations of the key sociodemographic variables and other covariates with delayed testing are presented in the eTable in Supplement 1.

## Discussion

Since the onset of the COVID-19 pandemic, researchers have demonstrated how inequities and health disparities are associated with infection rates, clinical outcomes, access to health care, testing, and vaccination rates. COVID-19 testing was initially a considerable challenge due to the availability of tests, which varied substantially across the US.^9^ However, once testing was more widely available, variability in symptomatic testing rates continued. In this cross-sectional study, we aimed to examine an underutilized metric by focusing on the timeliness of testing among a multicenter cohort of symptomatic individuals. This metric is important because time to testing may substantially alter personal and professional decisions to isolate, thus having the potential to impact the spread of infection. Second, delays in testing may also lead to false-negative results.^16^ Third, understanding the timeliness of testing allows us to compare subgroups for demographic disparities in testing as well as to identify organizational or structural constraints. To investigate potential disparities for organizational and structural constraints, we nested our research question within an ongoing, multicenter, vaccine effectiveness case-control study of HCP who have access to routine testing and largely understand the risks associated with infection.

One of our primary findings in this study was that race and ethnicity was associated with delayed COVID-19 testing in symptomatic HCP; non-Hispanic Black and non-Hispanic HCP of other races were more likely to have delayed testing compared with non-Hispanic White HCP. While the timeliness of testing is challenging to compare in the literature due to the scarcity of this metric, similar or related measures previously assessed include testing rates, vaccination rates, receipt of medications, and clinical outcomes. For example, in the general public, several studies^17,18,19,20,21,22,23,24,25,26,27^ observed racial disparities in COVID-19–related infections and outcomes, with lower testing, vaccination, and medication receipt among Asian and Black individuals compared with White individuals. Much of this disparity can be explained by inequities in our health care system and public health infrastructure; these factors are correlated with being underinsured, having fewer testing centers in areas that have higher proportions of Black individuals and other racial and ethnic minority groups, and/or lower rates of contact tracing.^26,28^ However, in our study of HCP that had adequate access to testing, we still observed disparities by race. When compared with other studies^29,30^ among HCP, this finding appeared to be consistent with the proxy measures of testing and vaccination. For example, several previous studies^29,30^ indicated either delayed vaccine uptake or lower receipt of vaccines by Black HCP compared with White HCP.

Even within a cohort of HCP, there may be additional barriers to timely testing. Implications of a positive COVID-19 test include facing stigma, disruptions in work and personal lives, and/or availability of paid leave.^31,32^ Other barriers were observed in vaccination hesitancy studies. For example, a previous study^29^ of vaccine receipt among HCP found that those with limited patient contact living in socially vulnerable areas were less likely to be vaccinated. The authors^29^ suggested that barriers such as the lack of perceived benefits from vaccination may serve as a barrier. We believe that this may be applicable to our findings; if the timeliness and importance of testing is not recognized in HCP, it will continue to be a barrier that propagates the existing racial disparities from the pandemic.

Lastly, differences in testing differed by job categories. Clinical HCPs with a graduate degree were less likely to have delayed testing compared with all other groups, including nonclinical HCPs with a graduate degree. In our sample, clinical HCPs with a graduate degree primarily consisted of physicians. Physicians likely experience expediated testing due to the increased risk and odds of COVID-19 infectivity and testing within 14 days.^33,34^ The differences in daily COVID-19 patient contact among physicians vs other groups contribute to self-risk assessment of COVID-19 infectivity. The direct patient contact that physicians have, including history taking and surgical and aerosolizing procedures, likely prompts physicians to test sooner to minimize nosocomial and community COVID-19 spread. Our study included academic institutions with equitable access to COVID-19 testing within the health care setting, therefore, this plausibly had minimal effect on occupational COVID-19 testing differences.

### Limitations

There are several limitations to this study. First, the HCP enrolled in this study were from academic medical centers with rigorous COVID-19 programs, trainings, and infection control programs. This may have influenced the timeliness of testing outcomes, such that any observed disparities or absence of findings may not be generalizable to HCP outside of this setting. Second, while the optimal timing for COVID-19 testing is when the onset of symptoms occurs, our binary classification of early (≤2 days) vs delayed (≥3 days) was based on the study investigators’ experiences of testing practices across sites and expected timeliness of testing from symptom onset. Third, as with any study recruiting volunteers, we suspect that there may have been potential selection bias by demographic factors that were associated with both recruitment and selection into the study and the outcome (eg, timeliness of testing). Individuals who opted into the study may have been more proactive, health-seeking individuals, thus representing a different population than those who opted out of the study. However, we anticipate that any potential selection bias would have resulted in a bias toward the null, suggesting that the observed findings may be attenuated from the true estimate. Fourth, symptoms and onset date were self-reported, which may have included some recall bias. While we expect that this could influence misclassification in terms of outcome, we do not expect differential misclassification of dates of symptom onset by demographic characteristics.

## Conclusion

In this cross-sectional study, we found that delayed testing was significantly higher among non-Hispanic Black HCP and non-Hispanic HCP of other races compared with non-Hispanic White HCP. In this study, we examined a novel metric of COVID-19 testing practices—timeliness of testing defined as completing COVID-19 testing within 2 days of symptom onset. Future efforts to investigate this metric in other populations (beyond HCP) and other settings may help identify trends and disparities in the community. Additionally, while we identified differences by race, we cannot fully understand why we observed this variability; these findings can be enhanced by qualitative work that investigates facilitators and barriers to timely testing.

## Notes

Supplement 1. **eFigure.** Histogram of Time to COVID-19 Testing From Symptom Onset **eTable.** Association Between Demographic Characteristics and Delayed Testing

Supplement 2.
PREVENT Nonauthor Collaborators

Supplement 3.
Data Sharing Statement
