# Supplementary material for: Race, Ethnicity, and Delayed Time to COVID-19 Testing Among US Health Care Workers
Source: JAMA Netw Open. 2024 Apr 10;7(4):e245697. doi: 10.1001/jamanetworkopen.2024.5697 (PMC11007575; doi:10.1001/jamanetworkopen.2024.5697)
Supplement: Supplement 1. — eFigure. Histogram of Time to COVID-19 Testing From Symptom Onset eTable. Association Between Demographic Characteristics and Delayed Testing [file jamanetwopen-e245697-s001.pdf]

Supplemental Online Content

Baymon DE, Vakkalanka JP, Krishnadasan A, et al; For the Preventing Emerging Infections through Vaccine EffectiveNess Testing (PREVENT) project. Race, ethnicity, and delayed time to COVID-19 testing among US health care workers. JAMA Netw Open. 2024;7(4):e245697. doi:10.1001/jamanetworkopen.2024.5697

eFigure. Histogram of Time to COVID-19 Testing From Symptom Onset
eTable. Association Between Demographic Characteristics and Delayed Testing

This supplemental material has been provided by the authors to give readers additional information about their work.

© 2024 Baymon DE et al. *JAMA Network Open*

eFigure. Histogram of Time To COVID-19 Testing From Symptom Onset

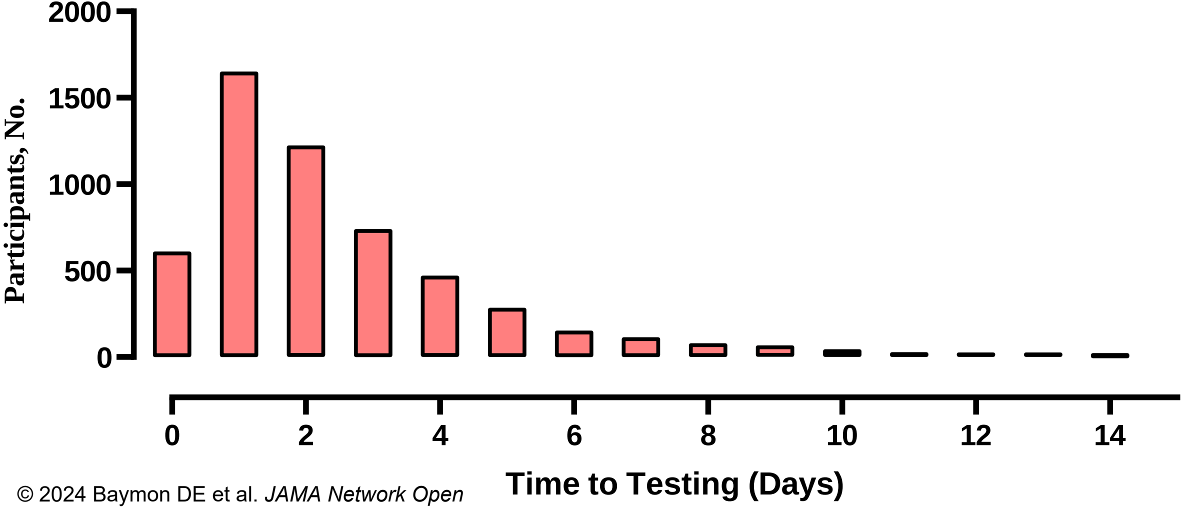

© 2024 Baymon DE et al. *JAMA Network Open*

eTable. Association Between Demographic Characteristics and Delayed Testing[1]

Individual - Demographic	aRR (95% CI)
**Sex (Ref = Female)**	
Male	1.00 (0.94-1.06)
**Age Group (Years) (Ref = 18-49 years)**	
25-34	0.99 (0.92-1.06)
35-49	0.97 (0.88-1.07)
50-64	1.08 (0.99-1.18)
**Race and Ethnicity (Ref = Non-Hispanic White)**	
Non-Hispanic Black	1.18 (1.10-1.27)
Non-Hispanic Asian	1.04 (0.89-1.22)
Non-Hispanic Other	1.17 (1.03-1.33)
Hispanic - White	1.03 (0.87-1.22)
Hispanic - Other	1.16 (0.99-1.36)
Other	0.58 (0.42-0.80)
**Job and Education Classification (Ref = Clinical, Graduate Degree)**	
Clinical - Some College/College/Technical	1.25 (1.11-1.40)
Non-Clinical - Graduate Degree	1.17 (1.03-1.32)
Non-Clinical - Some College/College/Technical	1.26 (1.13-1.41)
High school or Less	1.36 (1.09-1.69)
Unknown	1.06 (0.16-6.93)
**Clinical Characteristics**	
≥3 comorbidities total (Ref = ≤2 comorbidities)	1.18 (1.10-1.27)
Asthma (Ref = No)	1.07 (1.01-1.15)
Diabetes (Type I or II) (Ref = No)	0.86 (0.73-1.02)
Hypertension (Ref = No)	0.96 (0.86-1.07)
Smoking Status (Ref = Never smoked)	
Current smoker	0.99 (0.85-1.15)
Former smoker	0.96 (0.85-1.07)
Prefer not to answer	1.26 (1.14-1.40)
**COVID-19 Characteristics**	
Variant (Ref = Wild Type/Alpha)	
Delta	0.93 (0.85-1.02)
Omicron	0.81 (0.72-0.91)
Vaccination (Ref = None)	
Yes, 2-dose	0.99 (0.91-1.08)
Incomplete	0.91 (0.82-1.00)

[1] Also adjusted for study site (results not presented)

© 2024 Baymon DE et al. JAMA Network Open
