# Supplementary material for: Race, Ethnicity, and Delayed Time to COVID-19 Testing Among US Health Care Workers
Source: JAMA Netw Open. 2024 Apr 10;7(4):e245697. doi: 10.1001/jamanetworkopen.2024.5697 (PMC11007575; doi:10.1001/jamanetworkopen.2024.5697)
Supplement: Supplement 2. — PREVENT Nonauthor Collaborators [file jamanetwopen-e245697-s002.pdf]

Supplemental Online Content: Nonauthor Collaborators
*First name, last name, and suffix (if applicable) are required and will appear in PubMed.

*Group Name(s): PREVENT							
***First Name and Middle Initial(s)**	***Last Name**	***Suffix (eg, Jr, III)**	Academic Degrees	Institution	Location (city, state/province, country)	Role or Contribution, eg, chair, principal investigator	Group (if more than 1 Group listed in the byline) and/or Subgroup (eg, Steering Committee)
Howard A.	Smithline		MD, MS				
Lilly C.	Lee		MD, SM				
Stephen C.	Lim		MD				
Gregory J.	Moran		MD				
Mark T.	Steele		MD				
David G.	Beiser		MD, MS				
Brett	Faine		PharmD, MS				
Utsav	Nandi		MD MSCI				
Walter A.	Schrading		MD				
Brian	Chinnock		MD				
Anne	Chipman		MD, MS				
Megan	Fuentes		BS				
Frank	LoVecchio		DO, MPH				
Bradley	Clinansmith		BS				
Shannon	Landers		BA				
Alysia	Horcher		MPAS, PA-C				
Lisandra	Uribe		BS				
Kavitha	Pathmarajah		MPH				
Kye E.	Poronsky		MS				
Dean M.	Hashimoto		MD				
Monica	Bahamon		MPH				
Michelle	St. Romain		MD				
Efrat	Kean		MD				
Elizabeth	Krebs		MD,MSc				
Amy	Stubbs		MD				
Sara	Roy		MSCR				
Gregory	Volturo		MD				
Amanda	Higgins		MS				
James	Galbraith		MD				
James C.	Crosby		MD				
Mary	Mulrow		MA, MN				
Eva	Gonzalez		BA				
Ryan	Gierke		MPH				

Supplemental Online Content: Nonauthor Collaborators
*First name, last name, and suffix (if applicable) are required and will appear in PubMed.

***First Name and Middle Initial(s)**	***Last Name**	***Suffix (eg, Jr, III)**	Academic Degrees	Institution	Location (city, state/province, country)	Role or Contribution, eg, chair, principal investigator	Group (if more than 1 Group listed in the byline) and/or Subgroup (eg, Steering Committee)
Jennifer L.	Farrar		MPH				
Wei	Xing						
Yunmi	Chung		MPH				
Anna	Yousaf		MD				
Jennifer	Onukwube Okaro		MPH				
Glen R.	Abedi		MPH				
Sankan	Nyanseor		MPH				
Christopher K.	Watts		MPH				
