# Supplementary material for: Race, Ethnicity, and Delayed Time to COVID-19 Testing Among US Health Care Workers
Source: JAMA Netw Open. 2024 Apr 10;7(4):e245697. doi: 10.1001/jamanetworkopen.2024.5697 (PMC11007575; doi:10.1001/jamanetworkopen.2024.5697)
Supplement: Supplement 3. — Data Sharing Statement [file jamanetwopen-e245697-s003.pdf]

Data Sharing Statement

Baymon. Race, Ethnicity, and Delayed Time to COVID-19 Testing Among US Health Care Workers. *JAMA Netw Open*. Published April 10, 2024.
doi:10.1001/jamanetworkopen.2024.5697

Data
Data available: Yes
Data types: Deidentified participant data
How to access data: dbaymon@mgb.org
When available: With publication

Supporting Documents
Document types: Informed consent form, Statistical/analytic code
How to access documents: dbaymon@mgb.org
When available: With publication

Additional Information
Who can access the data: anyone requesting the data
Types of analyses: for alternative research analysis or statistical confirmation
Mechanisms of data availability: after approval of a proposal with a signed data access agreement and investigator support
